# In a simulated adult trauma patient, can pelvic binders be applied accurately by paramedics and HEMS paramedics? A pilot observational study

**DOI:** 10.29045/14784726.2021.6.6.1.23

**Published:** 2021-05-01

**Authors:** Samuel McCreesh

**Affiliations:** Yorkshire Ambulance Service NHS Trust

**Keywords:** emergency medical services, HEMS, pelvic binder, pelvic injury, pre-hospital, trauma

## Abstract

**Background:**

Pre-hospital treatment of suspected haemorrhagic pelvic fractures includes application of a purpose-made pelvic binder. Recent hospital studies identified poor accuracy of pelvic binder application, but there is little pre-hospital research to date.

**Methods:**

A pilot observational study was conducted in an NHS ambulance service to examine the accuracy of landmark identification and pelvic binder application. Paramedics and Helicopter Emergency Medical Service (HEMS) paramedics were recruited via an internal advert. Participants were asked to name and identify the landmarks (greater trochanters) on a simulated patient and apply the Prometheus pelvic splint. Participants read two clinical scenarios and indicated if they would apply a pelvic binder. Descriptive and inferential statistics were used in the analysis of results to compare performance between the two groups.

**Results:**

Twenty-six paramedics were recruited. A total of 92.3% (n = 12) paramedics and 100% (n = 13) HEMS paramedics verbalised the correct landmarks. A total of 23.1% (n = 3) paramedics and 61.5% (n = 8) HEMS paramedics identified the correct landmarks on both sides of the pelvis. A total of 15.4% (n = 2) paramedics and 61.5% (n = 8) HEMS paramedics applied the pelvic binder centrally over both greater trochanters. Clinical decision-making to apply a pelvic binder was largely in accordance with a local standard operating procedure.

**Conclusion:**

This study supports existing research highlighting cases of inaccurate pelvic binder placement. HEMS paramedics were more accurate than paramedics, but only 39% of all binders placed in the study were applied correctly. Frequent exposure to major trauma and familiarity with pelvic binders may have resulted in greater accuracy among HEMS paramedics. Further education and training around clinical assessment of the pelvis may improve the accuracy of pelvic binder application by all paramedics. This would subsequently improve the quality of patient care and ensure adequate haemorrhage control is maintained.

## Introduction

Pre-hospital treatment of suspected pelvic injuries has become a topical subject in recent years, as paramedics are increasingly held accountable for their clinical decision-making following the transition from historical training programmes to a higher education entry-level profession (College of Paramedics, 2017). Pelvic injuries account for 8% of all musculoskeletal injuries and are associated with other injuries in 90% of cases (Acharya & Forward, 2014). Pelvic fractures with associated haemorrhagic shock have mortality rates as high as 54% (Acharya & Forward, 2014), highlighting the importance of accurate assessment and early pre-hospital treatment. While historical outcomes from major trauma were poor, the 2012 reconfiguration of trauma services in England has resulted in significant improvements in treatment and survival rates (Moran et al., 2018). It is recommended patients with suspected pelvic fractures bypass local hospitals to a major trauma centre if it is within a one-hour isochrone (National Institute for Health and Care Excellence, 2016).

NHS ambulance services provide the statutory response to emergency incidents involving trauma (National Audit Office, 2017), often accompanied by a Helicopter Emergency Medical Service (HEMS) usually operated by air ambulance charities (Littlewood et al., 2010). In addition to rapid transport, specialist HEMS paramedics and physicians provide pharmacological and surgical interventions to patients with traumatic injuries beyond the scope of practice of most UK paramedics. Pelvic injuries occur in patients who have suffered a high energy impact mechanism of injury (Ward et al., 2018). Road traffic collisions account for two thirds of pelvic injuries, followed by motorcycle incidents and falls from height (Ward et al., 2018). Considering the relationship between pelvic fractures and other traumatic injuries, HEMS paramedics are frequently exposed to patients with these injury patterns.

Existing research has identified poor accuracy of pelvic binder placement among patients attending hospital with suspected pelvic injuries (Barnes et al., 2017; Bonner et al., 2011; Henning et al., 2018). There has been little research focusing solely on paramedic-led care, despite recommendations (Barnes et al., 2017), while studies about pelvic binder use in UK HEMS relate to physician-led treatment (Browne & Corfield, 2016; Yong et al., 2016). Considering the severe complications documented as a result of inadequate pelvic binder placement, such as pressure sores, tissue necrosis, inadequate pelvic stabilisation and worsening haemorrhage (Barnes et al., 2017; Coccolini et al., 2017), the author identified a need for further research relating to paramedic use of pelvic binders.

Treatment of a pelvic fracture with suspected haemorrhage includes the application of a purpose-made pelvic binder to stabilise the pelvis, promote clot formation and reduce internal haemorrhage (Scott et al., 2013). There are various pelvic binders in use across UK ambulance services (Henning et al., 2018) and individual services will often publish accompanying standard operating procedures (SOPs) relating to their application. The Prometheus pelvic splint (Prometheus Medical Ltd., 2016) is used within the author’s ambulance service and HEMS organisation and was therefore selected as the device of choice for this study. The aim of the study was to compare the performance of non-specialist paramedics (primarily operating on a double-crewed ambulance or rapid response vehicle) and HEMS paramedics in relation to correct anatomical landmarking and accurate pelvic binder application in a simulated adult trauma patient. In addition, adherence to a local SOP was tested. The researcher also intended to consider whether a further, large-scale study was feasible.

## Methods

A pilot observational study was conducted within a regional UK ambulance service. Yorkshire Ambulance Service NHS Trust covers a diverse range of densely-populated urban areas and sparsely-populated rural areas. Therefore, paramedics’ clinical exposure to trauma may vary widely across the region. HEMS paramedics were employed by the ambulance service and seconded to the HEMS charity; therefore, all participants were NHS employees. Prior to commencing the study, written consent was gained from the ambulance service research and development department, HEMS charity and HEMS operations manager. The research was conducted as part of an educational programme, therefore ethical approval was granted by the university ethics committee. An internal advert ran for three weeks during January 2019 in the ambulance service bulletin advertising for participants. A convenience sample was selected by the lead researcher in order of response. There were no restrictions upon length of experience in order to maximise the number of participants recruited. The study was open to participants from across the region, in the hope that data would be representative of practice across the entire region and to maximise the number of paramedics recruited. Participants were sent an information sheet by email and informed consent was gained prior to them taking part in the study.

The clinical grade and length of experience of each participant were recorded. The researcher read from a scripted observation schedule to standardise the information available to participants during scenarios and to avoid bias. A SimBodies^®^ adult male human patient simulator was utilised as the patient in all scenarios for consistency between participants and due to its highly realistic anatomical landmarks. The SimBodies^®^ manikin used in this study features deformity to the pelvis, a clinical sign of pelvic injury (Coccolini et al., 2017; Lee & Porter, 2007). Each participant was instructed to name the correct landmarks for application of a pelvic binder to a patient. Participants were asked to locate the landmarks on the patient and to mark them with a 20 mm circular black sticker. They were asked to apply the Prometheus pelvic splint to the patient, utilising the same techniques as their normal clinical practice. A non-clinical assistant was available to assist the participants, acting under the participant’s instruction only, to mimic the normal working practice of a paramedic and emergency care assistant or emergency medical technician. At the end, participants were asked to read two pre-written clinical trauma scenarios and answer yes or no to whether they would apply a pelvic binder to the patient. Local indications for application of a pelvic binder are ‘obvious open book pelvic fracture or suspected pelvic fracture with associated active bleeding following significant mechanism’. The scenarios were not formally validated but were peer-reviewed to gain expert consensus on the correct answers in accordance with the local SOP. Participants indicated on the consent form whether they wished to receive individual feedback after the study, to further aid their personal development and reflection.

**Figure 1:**
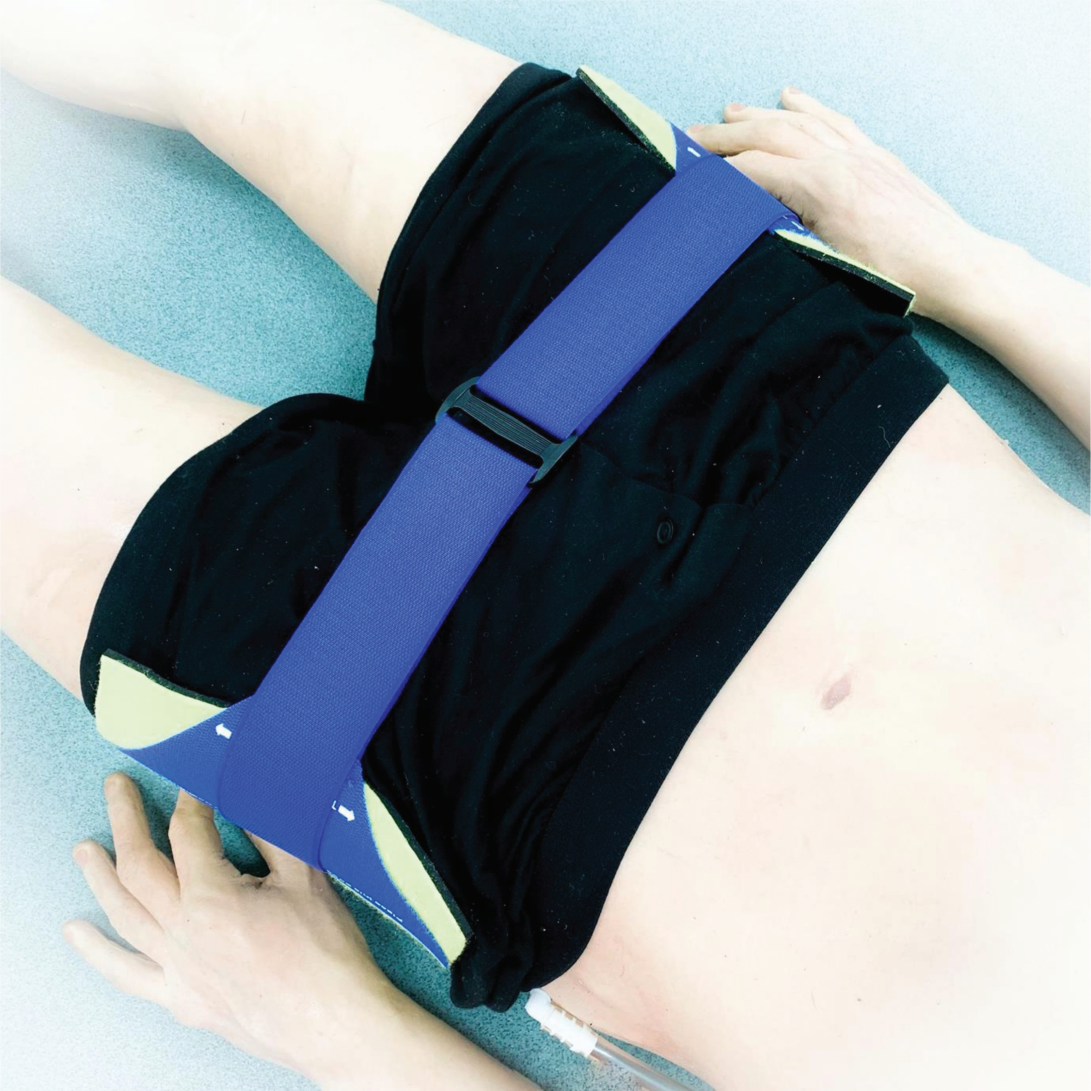
Figure 1. Correctly placed pelvic binder.

**Figure 2:**
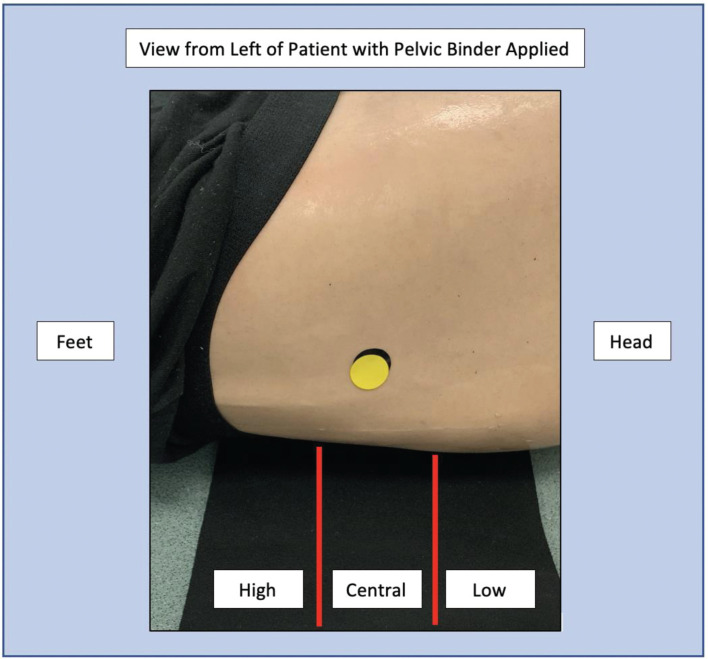
Figure 2. Example of pelvic binder placement marking.

Note: For example, the pelvic binder is positioned centrally in line with the greater trochanters (yellow marker). If the pelvic binder is slid down towards the feet of the patient, the top third of the binder (marked ‘low’) will be in line with the greater trochanters, resulting in a ‘too low’ placement. If the binder is positioned too high towards the head of the patient, the lower third of the binder (marked ‘high’) will be in line with the greater trochanters, resulting in a ‘too high’ placement.

Following each scenario, the researcher photographed the position of the pelvic binder from three angles. The pelvic binder placement was photographed from above before it was carefully unfolded to avoid any movement (Figure 1). The researcher identified the bony prominent markers of the greater trochanters on each side of the manikin with a 20 mm yellow sticker. This allowed the markers to compare the manikin’s landmarks with where participants had identified the landmarks. The placement of the pelvic binder was photographed from both sides to assess whether each side of the binder was applied centrally in line with the correct landmarks (Figure 2). To allow for a degree of tolerance, the landmark was considered correctly identified if any part of the participant’s sticker overlapped the marker’s sticker. The pelvic binder placement was marked ‘central’ if the greater trochanters were in line with the central third of the pelvic binder. If the sticker was outside of this region, the placement of the binder was marked as too high or too low. The results were anonymised and marked independently by the researcher and a senior critical care paramedic from another HEMS charity.

The research question sought to make comparisons between the two independent study groups (paramedics and HEMS paramedics). The significance level was defined at the 0.05 (5%) level in accordance with conventional standards (Freeman & Walters, 2006). Statistical analysis was performed using an independent samples t-test and Fisher’s exact test. A null hypothesis was formulated, assuming no differences between the groups.

## Results

Twenty-six paramedics were recruited: 13 paramedics and 13 HEMS paramedics (Table 1). The paramedics’ mean experience in practice as a paramedic was 3.26 years (2.88 standard deviation (SD)) compared to 12.8 years (3.98 SD) mean experience as a qualified paramedic in the HEMS paramedic group. A Kolmogorov-Smirnov test for normality showed the participant experience was normally distributed across each group. Statistical analysis with an independent samples t-test showed a statistically significant difference between paramedic and HEMS paramedic mean years of experience (p = < 0.001). A Fisher’s exact test showed a statistically significant difference between paramedic and HEMS paramedic pelvic binder placement (p = 0.041). Despite the differences between accuracy of landmark identification, this was not statistically significant.

**Table 1.** Experience of participants, accuracy of landmark identification and pelvic binder application by paramedics and HEMS paramedics.

	Paramedic	HEMS paramedic	p-value
**Experience (years) as a -paramedic – mean (SD)**	3.26 (2.88)	12.8 (3.98)	< 0.001
**Q1: Verbalised landmarks ‘greater trochanters’ – n (%)**	12 (92.3)	13 (100)	1.000
**Q2: Identified landmarks (both sides) – n (%)**	3 (23.1)	8 (61.5)	0.111
**Q3: Correctly applied pelvic binder (both sides) – n (%)**	2 (15.4)	8 (61.5)	0.041
**Scenario 1 pelvic binder applied – n (%)**	0 (0)	1 (7.7)	
**Scenario 2 pelvic binder applied – n (%)**	13 (100)	13 (100)	

HEMS = Helicopter Emergency Medical Service; SD = standard deviation.

**Figure 3:**
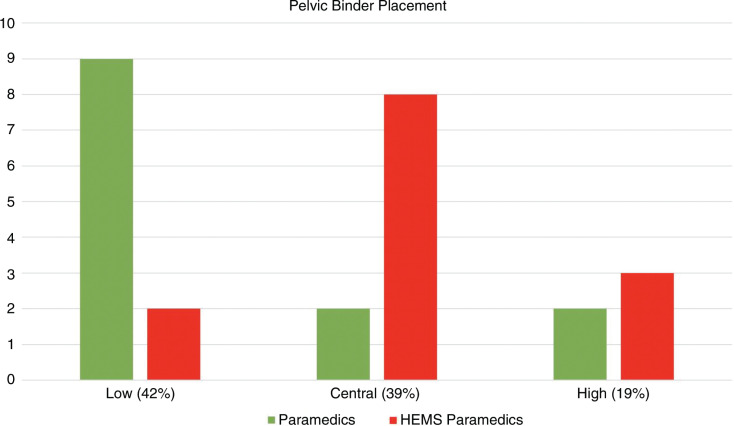
Figure 3. Pelvic binder placement.

A good standard of theoretical knowledge was demonstrated, as 92% of paramedics and 100% of HEMS paramedics correctly named the greater trochanters as the anatomical landmarks for the application of a pelvic binder. A total of 23% (n = 3) paramedics correctly identified the greater trochanters on both sides of the patient, compared to 61.5% (n = 8) of the HEMS paramedics. A total of 15% (n = 2) of paramedics and 61.5% (n = 8) of HEMS paramedics applied the pelvic binder centrally in line with the greater trochanters on both sides of the pelvis (Figure 3). While 61.5% (n = 8) of HEMS paramedics correctly identified the landmarks, only 46% (n = 6) of these went on to correctly apply the pelvic binder, while 15% (n = 2) applied the pelvic binder correctly on both sides despite not identifying the correct landmarks.

Two written clinical scenarios were used to test adherence to the SOP. Scenario 1 described an adult patient who had fallen 12 ft from height, who was haemodynamically stable with normal baseline observations, fully conscious and orientated, with no signs or symptoms of a pelvic injury. All of the paramedics (100%) stated they would not apply a pelvic binder. One HEMS paramedic indicated they would breach the SOP by applying a pelvic binder due to the mechanism of injury. Scenario 2 described an adult motorcyclist with polytrauma, a reduced Glasgow Coma Score, haemodynamic instability and signs of a pelvic fracture. All of the paramedics and HEMS paramedics (100%) indicated they would apply a pelvic binder to this patient, in accordance with the SOP.

Feedback was requested by 23 participants, suggesting this was a valued option. This was emailed to the participants, and included a detailed explanation of the research process, summary of results, overview of learning points and recommendations for future practice. Participants also received the opportunity to gain individual feedback relating to their performance.

## Discussion

### Accuracy of locating the anatomical landmarks

A majority of participants (96%) correctly named the greater trochanters as the anatomical landmarks for the application of a pelvic binder, demonstrating adequate theoretical knowledge of the landmarks for application of a pelvic binder. However, over half of all participants were unsuccessful in identifying the landmarks on both sides of the simulated patient, with only 23% of paramedics correctly identifying both landmarks. Performance was slightly better in the HEMS paramedics, with 61.5% identifying both landmarks.

One explanation could be the difficulty in clinicians locating the landmarks by physical palpation. It could be argued that utilising a simulated patient for the study is not as optimal as a human volunteer. To ensure consistency among participants it was necessary to utilise the same patient for all participants. Within the constraints of this small-scale pilot study, this could only be achieved using a manikin. The SimBodies^®^ manikin is an anatomically correct human simulator created from a human cast representing an adult male (Trauma FX, 2021), and was therefore considered as realistic as possible without using a real human volunteer. To examine whether the landmarks were identifiable, the researcher evaluated this among peers not involved in the research prior to commencing the study. No problems were encountered in locating the landmarks. Ease of locating the greater trochanters by palpation can vary by patient anatomy. Difficulty in locating landmarks on obese or bariatric patients has been cited as a barrier to accurate application of pelvic binders (Henning et al., 2018), therefore it is difficult to quantify this further.

Due to the deformity of the pelvis, the level of the greater trochanters is not symmetrical, as there is a 4 cm height difference between each side. This may have confused some participants, who failed to identify the deformity on inspection and palpation of the manikin and assumed that the landmarks would be symmetrical. Interestingly, the researcher witnessed some participants palpating one side of the pelvis, identifying the landmark and then placing the marker at the same level on the opposite side of the pelvis without palpating the second side to identify the second landmark. This may account for the four paramedics and three HEMS paramedics who identified only one correct landmark, highlighting the importance of accurate physical assessment. These results may demonstrate a lack of understanding of signs and symptoms of pelvic injury, or a lack of familiarity with pelvic deformity. The importance of examining the pelvis for these signs is well-documented in research and clinical guidelines (Brown et al., 2016; Coccolini et al., 2017; Ferris et al., 2015).

### Accuracy of pelvic binder placement

Several studies have demonstrated ineffective use of pelvic binders in reducing fractures and promoting clot formation, due to poor accuracy of placement (Barnes et al., 2017; Bonner et al., 2011; Henning et al., 2018). Data from this pilot study support the findings of existing research. A significant limitation of these studies is the lack of differentiation between pre-hospital and hospital placement of pelvic binders; therefore, this study sought to provide comparisons between two groups of paramedics, in an attempt to gain evidence relating to paramedic practice.

A pelvic binder should be applied centrally in line with the greater trochanters to achieve optimal reduction and stabilisation of pelvic fractures (Bakhshayesh et al., 2016). Unlike existing research, this study found that the majority of pelvic binders (42%) were placed too low (Figure 3). Existing studies have found more frequent cases of high pelvic binder placement compared to low placement. However, these studies relied on clinical imaging to assess accuracy, and may be subject to a higher degree of misinterpretation compared to direct inspection of the pelvic binder. A study by Henning et al. (2018) discovered 41% of pelvic binders were not placed correctly in a sample of 89 patients, as 31% were applied too high and 10% too low. High placement was most common in patients with a pelvic fracture diagnosis. While a number of different pelvic binders were included, the Prometheus pelvic splint had the highest rate of accuracy (67% central). However, they were also the smallest proportion of binders used (15/178). Another study (Bonner et al., 2011) examined pelvic binder placement in 173 patients within a military hospital setting and found that while 50% were placed centrally, 39% were placed too high and 11% too low. The study also found that high placement was inadequate in reducing fractures and managing haemorrhage compared to central placement. Similarly, while there was no breakdown of results by profession, the authors acknowledged the study included a large number of military healthcare professions and the results may be relevant to the civilian population.

It is beyond the scope of this study to examine the reasons for inadequate placement, nor were any data collected surrounding the different methods of application observed when participants applied the pelvic binder. The manufacturer’s instructions advise to fold the binder in half and apply in line with the greater trochanters, while performing a 10-degree patient tilt (Prometheus Medical Ltd., 2016). However, at the time of the study, a local SOP instructed paramedics to apply the pelvic binder using a method where the binder is inserted under the hollows of the patient’s knees and slid up under the pelvis. A disadvantage of this method is the potential for binders to be placed too low, if they are not slid up high enough under the pelvis in line with the greater trochanters. The author observed both methods of application during the study, but this was not formally recorded.

Inadequate pelvic binder placement as a result of alternative methods of application has previously been identified as a source of potential misplacement (Bonner et al., 2011). The ambulance service in this study has since amended its SOP to adopt the manufacturer’s instructions, but the use of the alternative method may have contributed to the number of low placed binders, highlighting the need to utilise the correct techniques when applying a pelvic binder. The author acknowledges the lack of guidance relating to application of pelvic binders where the pelvis is asymmetrical or where there is a difference in alignment of the greater trochanters. Existing guidance refers only to applying the binder centrally in line with the greater trochanters on both sides, which was therefore the criteria for correct placement in this study. Further research and guidance are required surrounding treatment of this presentation of injury.

### Adherence to a local standard operating procedure

This study aimed to gather data relating to clinician adherence to a local SOP for clinical indications to apply a pelvic binder. Anecdotally, the researcher’s experience is that paramedics often breach the SOP by applying pelvic binders according to mechanism of injury only, in the absence of clinical signs or symptoms of a pelvic injury. However, the results of this study show that the majority of paramedics and HEMS paramedics acted in accordance with the SOP. The sample recruited may have been more familiar with the SOP because they volunteered to participate in the study. Alternatively, the clinical scenarios may not have reliably tested this question, as they were not formally validated. Further research is required, including a wider range of scenarios, to evaluate decision-making according to different mechanisms of injury and clinical presentations.

### Experience of participants

A statistically significant difference in experience between the paramedic and HEMS paramedic groups’ mean years of experience was identified (p = < 0.001). A greater level of experience is expected among the HEMS paramedic group, as paramedics must gain significant clinical experience before they are eligible to apply for the HEMS specialist role. However, many of the ambulance paramedics recruited were newly-qualified or within the first few years of their career. In comparison, the most junior HEMS paramedic had eight years’ experience as a paramedic. Despite more recent pre-registration education and training, reduced clinical experience and exposure to trauma in the paramedic group may have attributed to lower levels of accuracy.

### Limitations

This was a small-scale pilot study and as such there are limitations. Firstly, it may be that paramedics and HEMS paramedics with an interest in trauma and pelvic injuries were more likely to participate, which may have resulted in bias. The paramedic sample size is too small to enable generalisations to be made, as the ambulance service in this study employs over 1000 paramedics. Therefore, the results cannot be considered representative of paramedic practice within this service. The presence of the researcher within the simulation may have resulted in an observer effect, affecting participants’ performance, as the majority of paramedics recruited were not previously known to the researcher. The clinical scenarios were not formally validated, whereas validation may have enhanced the content validity. The use of more written scenarios would have enabled the researcher to test a greater range of clinical presentations and situations within the SOP.

Use of a manikin rather than a human volunteer also has limitations, as many medical manikins are unrealistic in comparison with a human patient. The manikin was chosen to maximise realism of a patient with clinical signs of a pelvic injury (Trauma FX, 2021). Existing research supports the validity of high-fidelity simulation in replicating trauma scenarios and promoting clinical reasoning among paramedics (Abelsson et al., 2016). While use of a human volunteer may have allowed for even more realistic landmarks, this would not have tested paramedics’ ability to locate landmarks in a patient with signs and symptoms of a pelvic injury. A prospective study involving patients was outside the realms of this educational study.

## Conclusion

The results from this study suggest HEMS paramedics are more accurate when identifying the greater trochanters and correctly applying a pelvic binder to a simulated patient. Frequent exposure to major trauma and familiarity with the use of pelvic binders may suggest the reason for greater accuracy in this group. Adherence to a local SOP for applying a pelvic binder according to clinical presentation was similar between both samples of paramedics and HEMS paramedics. Further education and training around clinical assessment of the pelvis may improve the accuracy of pelvic binder application by all paramedics. This pilot study has several limitations, including a small sample size, low generalisability and limited validation. A further large-scale study is required to produce data representative of UK paramedic practice.

## Acknowledgements

The author would like to thank William Broughton (University of Hertfordshire) for his assistance as research supervisor for this study.

## Author contributions

SM is the lead researcher involved in this study and author of this article. SM acts as the guarantor for this article.

## Conflict of interest

None declared.

## Ethics

The study was approved by the University of Hertfordshire Ethics Committee protocol no. HSK/PGT/UH/03544.

## Funding

None.
